# Primary breast leiomyosarcoma: prognostic factors and treatment outcomes – a systematic review and case report (1969–2023)

**DOI:** 10.3389/fonc.2025.1662132

**Published:** 2025-10-30

**Authors:** Rui Guo, Weiwei Zhang, Xingli Ji, Xiaohu Liu, Lang He

**Affiliations:** ^1^ Department of Oncology, Cancer Prevention and Treatment Institute of Chengdu, Chengdu Fifth People’s Hospital (The Second Clinical Medical College, Affiliated Fifth People’s Hospital of Chengdu University of Traditional Chinese Medicine), Chengdu, China; ^2^ Department of Pathology, Chengdu Fifth People’s Hospital (The Second Clinical Medical College, Affiliated Fifth People’s Hospital of Chengdu University of Traditional Chinese Medicine), Chengdu, China; ^3^ Department of Imaging, Chengdu Fifth People’s Hospital (The Second Clinical Medical College, Affiliated Fifth People’s Hospital of Chengdu University of Traditional Chinese Medicine), Chengdu, China

**Keywords:** breast leiomyosarcoma, breast sarcoma, prognosis, overall survival, tumor size

## Abstract

**Objective:**

This study aims to investigate the prognostic factors and treatment outcomes of primary breast leiomyosarcoma (PBL). We present a contemporary case of postoperative recurrence and metastasis, and conduct a systematic review to comprehensively analyze all reported cases over the past 54 years.

**Method:**

We describe a 48-year-old female with primary breast leiomyosarcoma managed with multimodal therapy, including surgery, chemotherapy, radiotherapy, and immunotherapy. Additionally, a systematic review was conducted following the Preferred Reporting Items for Systematic Reviews and Meta-Analyses (PRISMA) 2020 guidelines. We searched multiple electronic databases for studies on PBL published between 1969 and 2023. Patient demographics, clinical characteristics, and treatment strategies were extracted from the eligible studies. Kaplan-Meier survival analysis was employed to assess overall survival (OS), and Cox proportional hazards regression models were used to evaluate prognostic factors, including age, tumor size, and treatment approach.

**Result:**

The systematic search identified 98 eligible studies, which collectively reported on 106 patients with PBL. The PRISMA 2020 flow diagram illustrated the study selection process. Among the patients, 86.8% were female, and 50.9% of tumors originated in the left breast. The mean pretreatment tumor diameter was 6.38 ± 4.98 cm. Surgical intervention was performed in 88.1% of cases, predominantly mastectomy. Survival analysis revealed a median OS of 18 months. Subgroup analysis demonstrated significantly shorter OS in patients aged ≤37 years at diagnosis or with tumors >7 cm (P<0.05). Multivariate Cox regression identified younger age at diagnosis as an independent predictor of poor prognosis (HR: 4.514, 95% CI: 1.146-17.784, *P* = 0.031).

**Conclusion:**

Surgical resection remains the cornerstone of treatment for PBL. Our findings, derived from a PRISMA-guided systematic review, highlight younger age at diagnosis as a significant adverse prognostic factor, underscoring the need for tailored therapeutic strategies for this high-risk subgroup.

## Introduction

Primary breast leiomyosarcoma, first described by Schmidt in 1887, is an exceedingly rare malignant mesenchymal tumor of the breast (1, 2). Breast sarcomas collectively account for approximately 1% of all breast malignancies, encompassing various histological subtypes including leiomyosarcoma, fibrosarcoma, angiosarcoma, and lymphosarcoma (3, 4). As a distinct entity, primary breast leiomyosarcoma predominantly affects postmenopausal women and demonstrates an intermediate prognosis - generally more favorable than other breast sarcomas but less favorable than epithelial breast carcinomas (5, 6). Despite numerous case reports in the literature, no standardized treatment protocol has been established for breast leiomyosarcoma.

Notably, while multiple case reports and limited series analyses have been published until 2024, a comprehensive systematic review incorporating survival analysis and prognostic factor evaluation of all reported cases over the past 54 years remains lacking. Therefore, we shared the diagnosis and treatment process of a 48-year-old female case of postoperative recurrence and metastasis of primary breast leiomyosarcoma, and conducted a literature review and secondary analysis of all case reports on primary breast leiomyosarcoma in the past 54 years.

## Materials and methods

### Study design

Given the absence of standardized treatment protocols for primary breast leiomyosarcoma, current management strategies are largely extrapolated from sarcoma therapies in other anatomical sites. To systematically characterize primary breast leiomyosarcoma, features and treatment outcomes, we conducted a comprehensive literature review of studies published between 1969 and 2023. Eligible publications in English and Chinese were identified through database searches, rigorously screened using predefined inclusion/exclusion criteria, and analyzed to evaluate associations between age, tumor size, and overall survival (OS).

### Eligibility criteria and research question

This systematic review was conducted to address the following question: “What are the prognostic factors and treatment outcomes for patients with primary breast leiomyosarcoma?” The eligibility criteria were structured using the PICOS framework: (1) Population: Patients of any age or gender with a histologically confirmed primary breast leiomyosarcoma. (2) Exposure: The diagnosis of primary breast leiomyosarcoma. (3) Comparators: Not applicable. (4) Outcomes: The primary outcome was overall survival (OS), defined as the time from diagnosis to death from any cause. Secondary outcomes included patient demographics, tumor characteristics (size, location), treatment details (surgery, chemotherapy, radiotherapy), and recurrence. (5) Study Designs: All published case reports, case series, and observational studies reporting on primary breast leiomyosarcoma were eligible for inclusion.

### Search strategy

Two independent reviewers (R.G. and W.Z.) performed a systematic search of PubMed, Web of Science, China National Knowledge Infrastructure (CNKI), and Wanfang Database for studies published from inception until December 31, 2023. The search strategy utilized the key terms “breast” AND “leiomyosarcoma” across all fields. The overall search strategy was (1) breast (all fields) and (2) leiomyosarcoma (all fields). Searches in electronic databases combined the terms 1 and 2. The complete, database-specific search strategies, including all keywords and Boolean operators, are provided in **Supplementary File 1**.

### Study selection criteria

The study selection process was conducted independently by the same two reviewers (R.G. and W.Z.). The reviewers independently screened the titles and abstracts of all retrieved records against the eligibility criteria. Studies that clearly did not meet the criteria were excluded. The full texts of all records that appeared relevant or whose eligibility was uncertain based on the title/abstract were retrieved. The same two reviewers then independently assessed these full-text articles for final inclusion. The following exclusion criteria were applied: (i) cases without a pathologically confirmed diagnosis of primary breast leiomyosarcoma; (ii) cases with missing critical information on age, tumor size, or survival outcomes; (iii) literature for which the full text was unavailable.

At both screening stages, any disagreement between the two reviewers regarding the inclusion or exclusion of a study was first addressed through discussion. If a consensus could not be reached, the final decision was made by a third senior reviewer (L.H.).

### Data extraction and management

To ensure the accuracy and consistency of data collection, a standardized data extraction form was developed *a priori*. The following data were extracted from each included study: first author, publication year, patient age and sex, tumor size and location, treatment modalities (surgery, chemotherapy, radiotherapy), and survival outcomes (overall survival (OS) time, status).

The data extraction was performed independently by two reviewers (R.G. and W.Z.) using this standardized form. To minimize errors and confirm data accuracy, the two reviewers cross-checked each other’s completed extraction forms. Any discrepancies or uncertainties in the extracted data were identified and then resolved by jointly reviewing the original source document. In cases where critical data (e.g., specific treatment details or exact survival times) were ambiguous or missing from the published report, we attempted to contact the corresponding authors via email to obtain clarification. No automation tools were used in the data collection process.

### Outcomes

The primary outcome of this systematic review was OS, defined as the time interval from the date of pathological diagnosis to death from any cause or the date of last follow-up for surviving patients. Secondary outcomes included key tumor characteristics (size, location, lymph node or vascular invasion status) and primary treatment modalities (surgery, chemotherapy, radiotherapy).

Regarding data completeness for each outcome, we sought the most definitive result available in each study. For OS, this meant extracting the final survival status at the longest reported follow-up time for each case, rather than survival rates at multiple pre-specified time points, due to inconsistent reporting across the included literature.

### Risk of bias assessment

The methodological quality of the included case reports and case series was assessed using the respective critical appraisal tools from the Joanna Briggs Institute (JBI). Two reviewers (X.J. and X.L.) independently conducted the assessments. If a consensus could not be achieved, a third senior reviewer (L.H.) was consulted to make the final decision. Given the retrospective and descriptive nature of the majority of included studies, which often lacked detailed reporting on specific criteria (e.g., unambiguous description of diagnostic criteria or follow-up schedules), the overall quality was variable. The primary aim of this assessment was to transparently characterize the strengths and limitations of the available evidence base rather than to exclude studies.

### Statistical analysis

Demographic and clinical characteristics of the study cohort, including mean age and tumor diameter, were analyzed using descriptive statistics. Continuous variables are presented as mean ± standard deviation. To evaluate survival outcomes, Kaplan-Meier (KM) analysis was performed to estimate median OS in all eligible patients. Survival distributions between subgroups were compared using log-rank tests. Univariate and multivariate Cox proportional hazards regression models were employed to assess the prognostic significance of key variables, including tumor characteristics (size and location), demographic factors (age and gender) and treatment modalities. These analyses were conducted to identify independent predictors of survival outcomes in breast leiomyosarcoma patients. All statistical tests were two-sided, with *P* < 0.05 considered statistically significant.

## Results

### Case presentation

**Figure 1:**
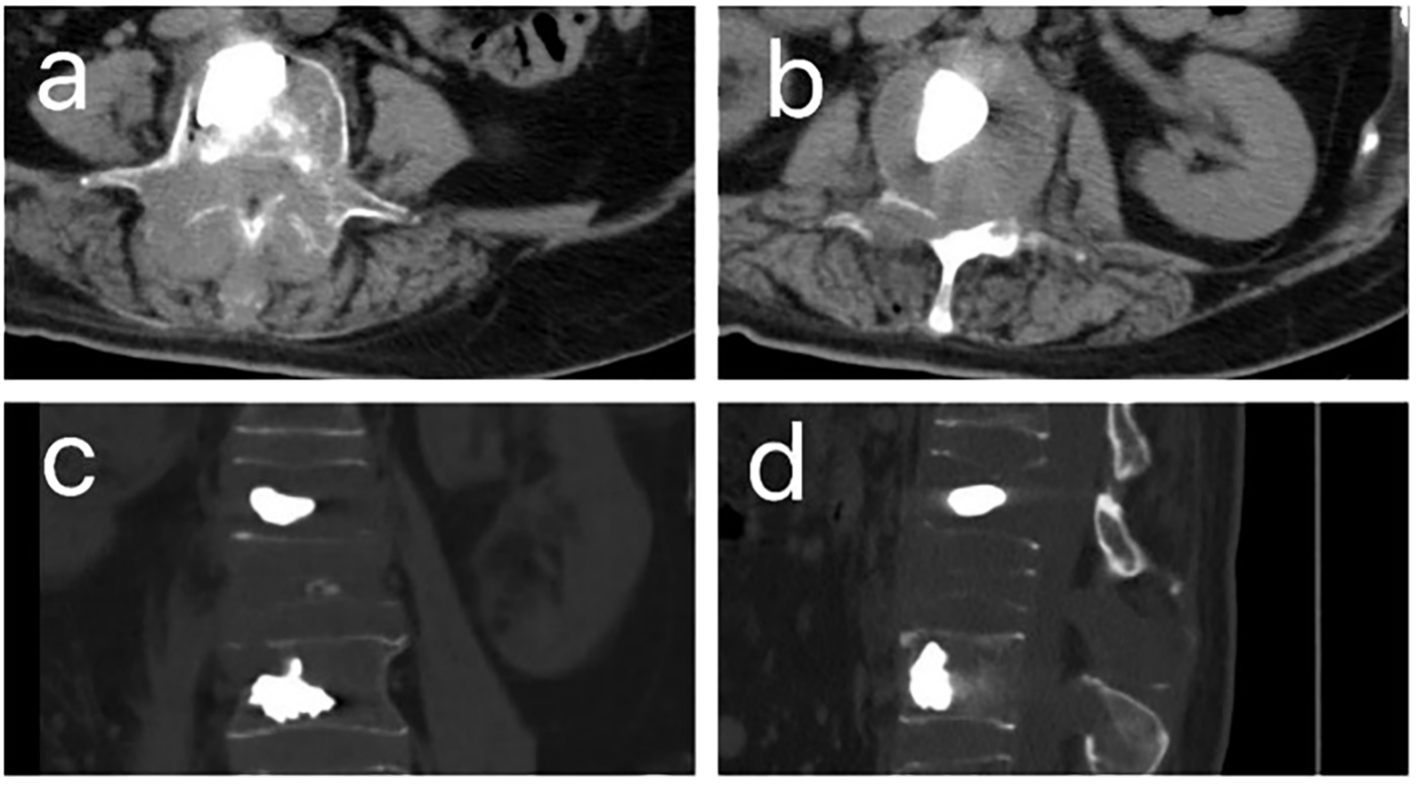
CT images after percutaneous kyphoplasty (PKP) of patient. **(A, B)**. The horizontal plane CT image of patient after PKP. **(C)**. The coronal CT images of patient after PKP. **(D)** The sagittal CT images of patient after PKP.

A 48-year-old woman presented with a 2×3 cm right breast mass discovered during routine physical examination. Following simple lumpectomy at a local hospital, pathological examination confirmed primary breast leiomyosarcoma (**Figure 1**), which was subsequently verified by our institutional review. The patient received no adjuvant therapy postoperatively. Two years later, she developed progressive lower back pain refractory to non-steroidal anti-inflammatory drugs (NSAIDs), accompanied by lumbar stiffness and ambulatory difficulty. Initial lumbar magnetic resonance imaging (MRI) demonstrated compression fractures without definitive intervention, and her symptoms progressively worsened to complete mobility impairment.

**Figure 2:**
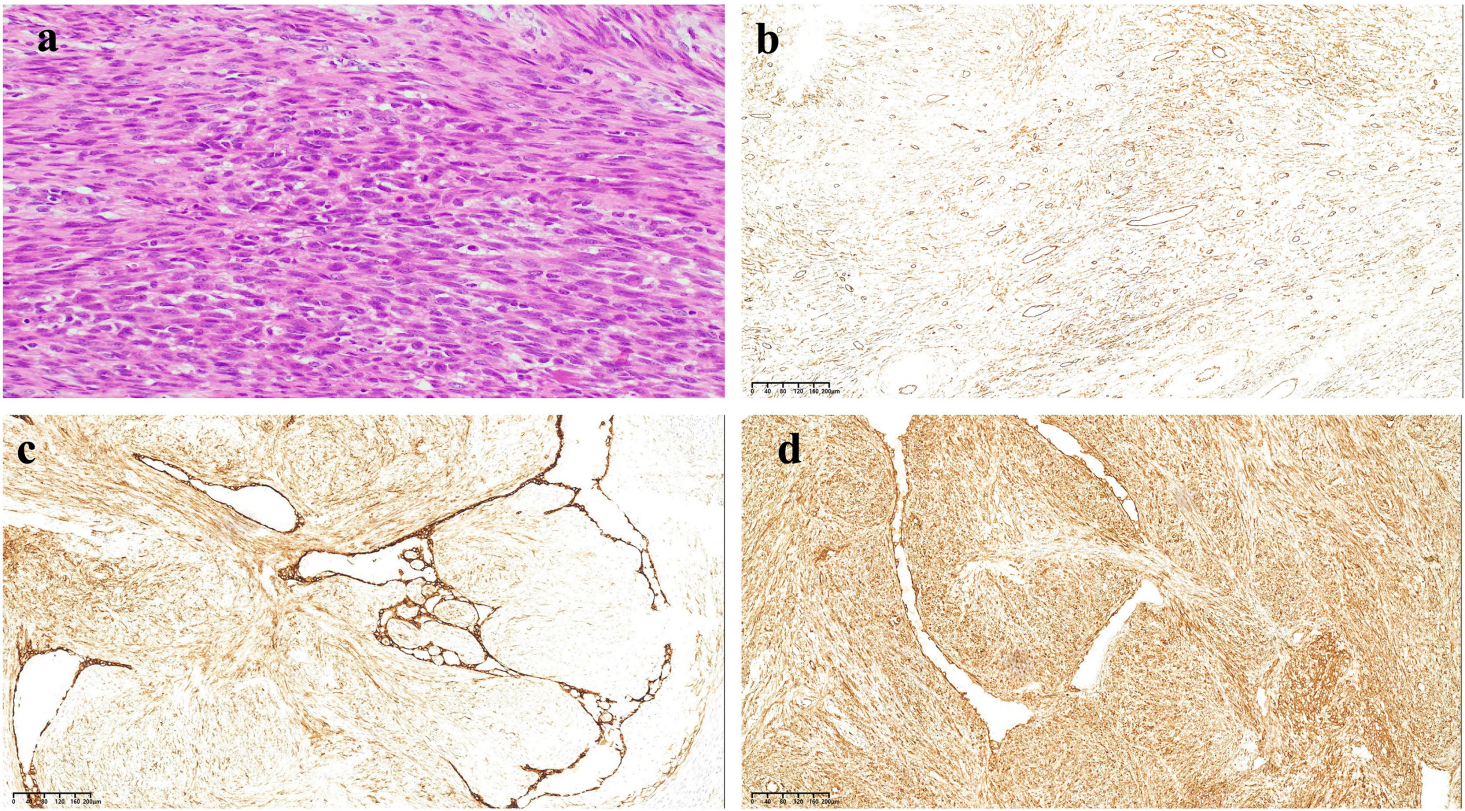
HE and immunohistochemical staining results of tumor tissue. **(a)** HE staining results show the tumor cells were spindle-shaped, infiltrative, and interlaced, with cigar-like nuclei, eosinophilic cytoplasm, perinuclear vacuoles, and has moderately anisotropic, nuclear schizophrenic and atypical nuclear schizophrenic images. **(b)** The tumor cells were positive for CD34 (×200). **(c)** The tumor cells were positive for CD10 (×200). **(d)** The tumor cells were positive for SMA (×200).

Ten months following symptom onset, repeat MRI revealed multiple thoracolumbar pathological fractures with posterior element destruction. The patient was subsequently referred to our orthopedic service with significant functional impairment (Eastern Cooperative Oncology Group Performance Status (ECOG) score is 3, bedbound status). She underwent C-arm guided percutaneous kyphoplasty (PKP) with biopsy of lumbar vertebrae 2 and 4 under local anesthesia, which demonstrated extensive osteolytic destruction involving the laminae, pedicles, and vertebral bodies. Notably, lumbar vertebrae 3 showed near-complete bony obliteration (**Figure 2**). Histopathological analysis confirmed metastatic breast leiomyosarcoma, supported by characteristic immunohistochemical profile: positive for smooth muscle actin (SMA), cluster of differentiation (CD) 34, and CD10, negative for Desmin and CD117, and with a low proliferative index (Ki-67 5-10%).

The patient came to our department for further treatment after the surgery. CT scan indicates that the patient only has bone metastasis and no recurrence or metastasis in other areas. Considering the extremely high risk of paraplegia, after sufficient communication with the patient and their family, IMRT technology was used to complete lumbar palliative radiation therapy. The radiation dose prescription was 95% DT P-GTVm1 = 6000cGy/30F/200cGy, P-GTVm2 = 4000cGy/20F/200cGy, P-CTV=3600cGy/20F/180cGy. After radiotherapy, 6 cycles of pembrolizumab combined with albumin bound paclitaxel and carboplatin were performed. The specific usage was pembrolizumab 200mg, albumin paclitaxel 260mg/m^2^, carboplatin AUC = 5 on day 1 every three weeks. After 6 cycles of treatment, the patient chose pembrolizumab immune maintenance therapy, administered every 3 weeks at a dose of 200mg each time, while also receiving bisphosphate to prevent bone related events. After treatment, the patient did not experience any further lumbar pain and returned to normal daily activities. The ECOG score was 1 point.

One year later, the patient began to experience back pain, which gradually worsened. Complete CT and MRI examinations revealed metastatic tumors in multiple vertebral bodies and some ribs of the thoracic and lumbar vertebrae, of which the thoracic 10 vertebral bodies were metastatic and invaded the spinal cord. Considering the progression of the tumor, palliative radiotherapy for the thoracic 10 vertebrae was performed at a dose of 95% DT P-GTVm=4500cGy/15F/300cGy, P-CTV=3000cGy/15F/200cGy. At the same time, 6 cycles of pembrolizumab combined with systemic intravenous chemotherapy were performed again. The chemotherapy regimen was pembrolizumab (200 mg on day 1), epirubicin hydrochloride (70 mg/m^2^ on day 1), ifosfamide (2000 mg/m^2^/day on day 1 to day 5), and mesna europrotection (400 mg/m^2^/day on day 1 to day 5). At present, the patient’s living condition is good, and as of the last follow-up, the total survival time of the patient is as long as 4 years.

### Study selection flowchart

**Figure 3:**
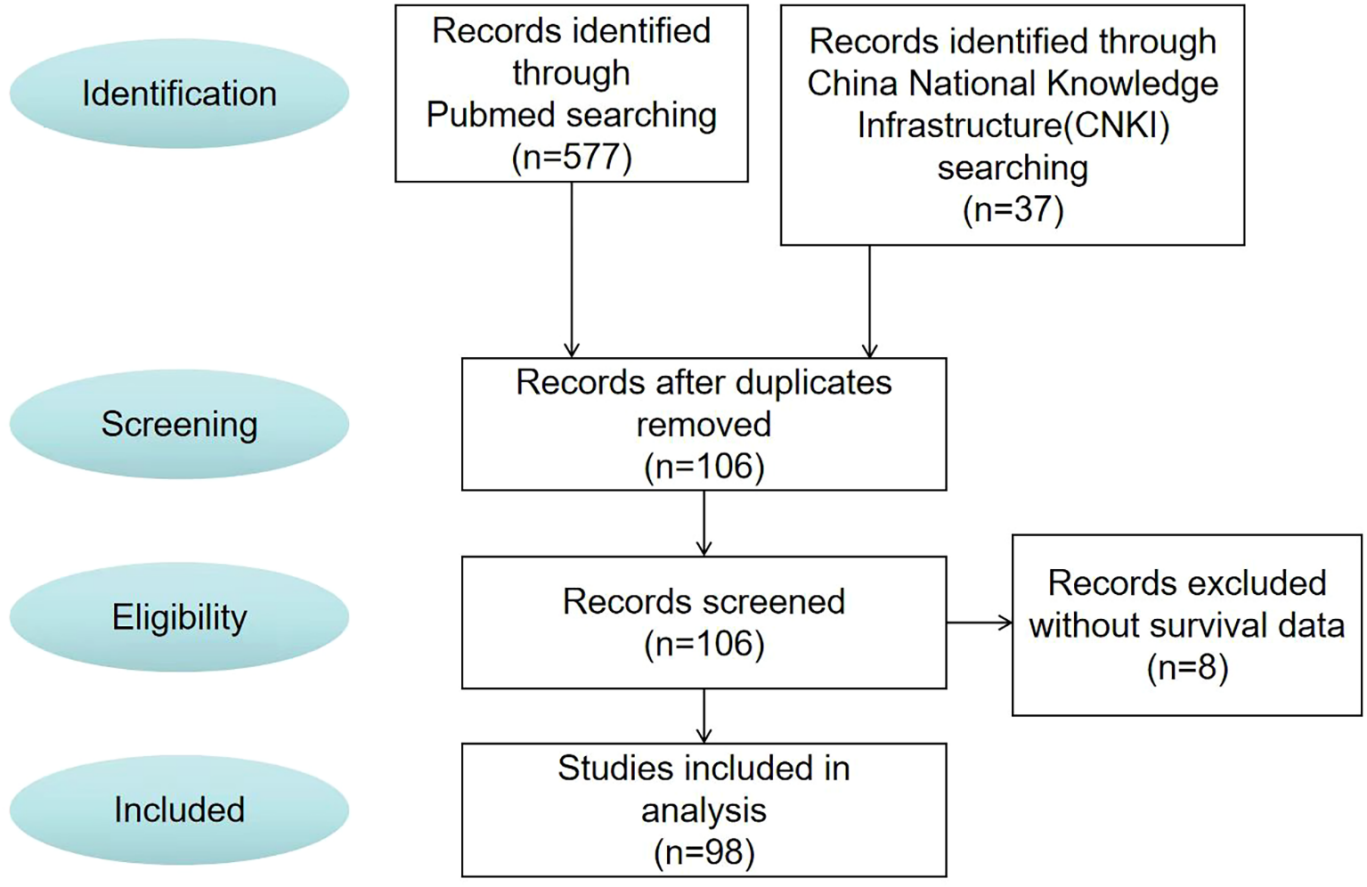
Paper selection flowchart.

After the screening process (**Figure 3**), 98 papers were deemed to meet our system evaluation criteria. **Table 1** lists the data of 106 cases in 98 articles. However, through further information extraction and screening of the literature, a total of 98 patients were included in subsequent survival analysis and Cox multivariate risk analysis.

**Table 1:** The clinical data of 106 cases.

No.	Author/s (year of publication)	Age (years)	Gender	Tumor location	Tumor size (cm)	Mitoses/10 HPF	Lymph node/ vascular invasion	Treatment	Metastasis	Outcome and follow-up
1	Crocker (1969) (7)	51	M	R	5	Common	NA	RM	NET	Alive, 7 months
2	Haagensen (1971) (8)	77	F	L	8	Frequent	NA	SM	NET	Alive, 14 years
3	Pardo-Mind 'an(1974) (9)	49	F	L	7	16	Yes	SM	NET	Alive, 6 months
4	Barnes and Pietruszka (1977) (10)	55	F	L	3	10	NA	SM	NET	Died, 4 years and 4 months later due to basilar artery thrombosis
5	Hernandez (1978) (11)	53	M	L	4	15	NO	MRM	NET	Alive, 14 months
6	Chen, Kuo and Hoffmann (1981) (12)	59	F	L	5.6	3	NO	SM	Hepatic metastasis	Alive, 15 years
7	Callery, Rosen and Kinne (1984) (13)	56	F	NA	2	NA	NA	SM	NET	Alive, 39 months
8	Callery, Rosen and Kinne (1984) (13)	54	F	NA	3	NA	NA	SM	NET	Alive, 53 months
9	Gobardhan (1984) (14)	50	F	L	9	5	NA	MRM	NET	Alive, 2 years
10	Yatsuka et al. (1984) (15)	56	F	L	1.5	21	NO	RM	NET	Alive, 4 years 7 months
11	Nielsen (1984) (16)	24	F	R	1.5	2	NO	Excision	Local recurrence and systemic recurrence (brain, skin, thyroid, kidney)	Died, 20 years later
12	Yamashina (1987) (17)	62	F	L	1	24	NA	RM	NET	Alive, 2 years 2months
13	Arista-Nasr et al. (1989) (18)	50	F	R	4.5	4	NA	Excision	Local recurrence	Alive, 6 years
14	Alessi and Sala (1992) (8)	62	M	R	NA	1	NA	Excision	Local recurrence	Alive, 6 years
15	Lonsdale and Widdison (1992) (19)	60	F	L	2	10	NO	SM	Local recurrence	Alive, 3 months
16	Parham et al. (1992) (20)	52	F	L	3	29	NA	SM	Local and systemic recurrence (brain, lung)	Alive, 6 months
17	Waterworth et al. (1992) (21)	58	F	L	4	10	NO	Excision	NET	Alive, 1 year
18	Wei (22)	36	F	L	4	NA	NA	MRM	Systemic recurrence (brain, bone), left breast	Died, 14 months later
19	Boscaino et al. (1994) (23)	56	F	R	2.5	2	NO	MRM	Local recurrence	Alive, 9 years
20	Boscaino et al. (1994) (23)	45	F	L	2.2	2	NO	WLE	Local recurrence	Alive, 40 months
21	Falconieri et al. (1997) (24)	83	F	R	6x5x5.5	20	NO	MRM	NET	Alive, 10 months
22	Falconieri et al. (1997) (24)	86	F	R	8x7x6	11	NO	SM	NET	Alive, 8 months
23	U ğras , et al. (1997) (25)	47	F	R	2	3	NO	Excision + SM	NET	Alive, 18 months
24	Gonzalez-Palacios F(1998) (26)	62	F	L	3	10	NA	SM	NET	Alive, 17 years
25	Gupta et al. (2000) (27)	80	F	L	7x5	8	NO	MRM	NET	Alive, 2 years
26	Hussien et al. (2001) (8)	49	F	R	2	≤12	NO	MRM	NET	Alive, 18 months
27	Szekely et al. (2001) (28)	73	F	R	4.5	20-22	NO	MRM	NET	Alive, 1 year
28	Kusama et al. (2002) (29)	55	F	L	1	10	NO	MRM	Local recurrence, systemic metastasis (lung, bone)	Alive, 4 years 8 months
29	Shinto et al. (2002) (30)	59	F	L	12	19	NO	MRM	Local and systemic recurrence (lung)	Alive, 8 months
30	Liang et al. (2003) (31)	25	F	L	4	5	NA	WLE	NET	Alive, 32 months
31	Markaki et al. (2003) (32)	42	F	R	10x4x14	50	NO	MRM	NET	Alive, 3 years
32	Markaki et al. (2003) (32)	65	F	L	5x1	10	NA	Excision	NET	Alive, 18 months
33	Jun Wei et al. (2003) (33)	52	F	R	1.5	22	NA	Excision	NET	Alive, 3 months
34	Adem (2004) (34)	67	F	NA	2	NA	NA	Excision	Local and systemic recurrence	Died, 7 months later
35	Adem (2004) (34)	55	F	NA	4	NA	NA	Mastectomy	Systemic recurrence	Died, 77 months later
36	Lee et al. (2004) (35)	44	F	NA	3	6-12	NA	SM	NET	Alive, 13 months
37	Lee et al. (2004) (35)	52	F	NA	4.5	6-12	NA	SM	NET	Alive,17 months
38	Munitiz et al. (2004) (36)	58	F	R	4	14	NO	MRM	NET	Alive,12 months
39	Stafyla, Gauvin and Farley (2004) (37)	53	F	L	23	NA	NO	MRM	NET	Alive, 2 years
40	Jayaram, Jayalakshmi and Yip (2005) (38)	55	F	R	12	40	NO	MRM	Local recurrence	Alive, 2 months
41	Gupta (2007) (39)	37	F	R	8x6	15	NO	WLE	NET	Alive, 36 months
42	Ende (2007) (8)	48	F	L	1.2	0	NA	Excision	NA	NA
43	De la Pena and Wapnir (2008) (40)	50	F	L	3.5x1.4x2.8	Few	NA	SM	NET	Alive, 11 months
44	Wong et al. (2008) (41)	52	F	L	1.5x1.10.7	7	NA	SM	NET	Alive, 4days
45	Cobanoglu et al. (2009) (42)	64	F	L	3.6	≤12	NO	MRM	NET	Alive, 22 months
46	Boehm et al. (2010) (43)	62	M	R	4.6x3.5	4	NA	MRM	NET	Alive, 24 months
47	Kamio (2010) (43)	46	F	L	0.5	2-8	NA	SM	NET	Alive, 8 years 4 months
48	Masannat Y et al. (2010) (43)	59	M	R	1.8x1.3	NA	NA	SM	NET	Alive, 26 months
49	Sandhya et al. (2010) (43)	54	F	L	7x7	6	NO	MRM	NET	Alive, 1 year
50	Fujita et al. (2011) (43)	18	F	R	7.2	10	NO	SM	NET	Alive, 5 years
51	Oktay and Fikret (2011) (43)	44	F	L	3.5	Few	NA	Excision	NET	Alive, 12 months
52	Nagao et al. (2011) (8)	61	F	R	3	>10	NA	WLE	NET	Alive, 18 months
53	Karabulut, Akkaya and Moray (2012) (43)	48	F	R	10x9x6	Frequent	NO	MRM	NET	Alive, 1 month
54	Rane, Batra and Saikia (2012) (43)	19	F	L	8	20-25	NA	Excision	NET	Alive, 3 years
55	Pai and Yoon (2013) (43)	46	F	L	7x6x6.5	>10	NA	MRM	Lung	Alive, 3 months
56	Yener and Aksoy (2013) (43)	44	F	L	3.5	Few	NA	Lumpectomy	NET	Alive,12 months
57	Amaadour et al. (2013) (43)	44	F	R	9.2x7.6x6	6	NA	Palliative CT	lung and abdominal wall	Died, 1 month later
58	Basset et al. (2014) (43)	20	F	L	3	High	NO	Excision + MRM	NA	NA
59	Guedes et al. (2014) (8)	46	F	R	1.6x1	3	NA	Excision	NET	Alive, 1 year
60	Agrawal, Garg and Pandey (2015) (8)	40	F	R	9x9x8	Frequent	YES	MRM	NET	Alive, 1 year
61	Agrawal, Garg and Pandey (2015) (8)	70	F	L	8x7x6	NA	NO	MRM	NET	Alive, 1 year
62	Sokolovskaya et al. (2014) (43)	58	F	R	15x9x13	NA	NA	MRM	Multiple bone and lung	Alive, 2 years
63	Kim et al. (2015) (43)	51	F	R	4x3x4	15	NA	Excision	NET	Alive, 5 years
64	Tajima, Koda and Fukayama (2015) (43)	50	F	L	4.8x4.5x4.2	6	NO	MRM	NET	Alive, 6 months
65	M’rabet et al. (2017) (43)	40	F	L	6	NA	NO	MRM + RT	NET	Alive, 8 years
66	Arsalane et al. (2017) (8)	68	M	R	8x9	9-10	NO	MRM	NET	Alive, 9 months
67	Testori et al. (2017) (4)	62	F	L	0.3x0.15	Up to 5	NO	Breast conservative surgery	NA	NA
68	Singh, Sharma and Goyal (2017) (44)	48	F	R	16x10	Numerous	NA	MRM + RT + CT	NET	Alive
69	Lee and Lee (2017) (43)	49	F	L	6x8	≤18	NA	Palliative mastectomy and CT	Lung	Died 4 months later due to sudden steep decrease in blood pressure
70	Villegas et al. (2018) (8)	48	M	L	8x5	NA	NO	SM	NET	Alive, 1 month
71	Amberger et al. (2018) (43)	20	F	L	3	30	NO	Excision + MRM	Lung	Alive, 3 years
72	Ilyas et al. (2020) (8)	52	F	L	6	2-50	YES	SM	NET	Alive, 1 year
73	Liu et al. (2020) (8)	28	F	L	1.6x0.9	10	NA	WLE	NET	Alive, 1 year
74	Kumar et al. (2020) (8)	53	F	R	8x6	Frequent	NA	WLE	Systemic recurrence (lung, renal, and skeletal)	NA
75	Horton et al. (2020) (8)	61	F	R	1.6	NA	NO	WLE	NA	NA
76	Bürger et al. (2020) (8)	54	F	R	3	Up to 3	NO	Breast conservative surgery	NET	Alive, 24 months
77	Ely Cheikh et al. (2021) (8)	65	M	RL	7	15	NA	L: WLE R:SM + RT	NET	Alive, 11 months
78	Rina Masadah (2023) (8)	30	F	L	12x8	>10	NO	WLE	NET	Alive, 8 months
79	Yan cunli (2015) (45)	27	F	R	4x2	NA	NO	SM+CT	NET	Alive, 12 months
80	Hong jiafan (2013) (46)	65	M	R	2x1.8x1.5	>5	NO	RM	NET	Alive, 3months
81	Yan Juan (2014) (47)	42	F	L	7x7x7	0	NO	SM	NET	Alive, 12months
82	Xiao Mingzhen (2013) (48)	29	F	R	7x6x4	3	NO	SM	NET	Aline 5months
83	Wang Na (2012) (49)	39	F	R	20x20x20	>5	NO	SM	NET	Alive, 24 months
84	Chen kaixing (1995) (2)	55	M	R	5x4x3	>10	NO	MRM	NA	Dead, 5months later
85	Bao luping (2003) (50)	57	M	R	4x3.5x3	>5	NO	SM+RT	NET	Alive, 3 years
86	Zhang Jian (2010) (51)	73	M	L	3.5x3x3	3	NO	SM	Systemic recurrence	Dead, 7 years later
87	Jiang xiaojun (2006) (52)	58	F	R	7x6x5	NA	NA	SM	NA	NA
88	Liu Xiaojun (2002) (53)	50	M	L	18x15x9	1.6	NO	SM+RT	NA	Alive, 12 months
89	Yan Peng (2005) (54)	73	M	L	3.5x3	NA	NO	SM	NET	Alive, 7 years
90	Yan Peng (2005) (54)	48	F	L	15x15	NA	NO	SM	Systemic recurrence (lung)	Dead, 1 year later
91	XuTian wen 1999 (55)	40	F	R	4x3.5x3	6	NA	RM	NET	Alive, 8 years
92	Wu Tianhui (2002) (56)	38	F	R	22x22x12	>10	NO	RM	NET	Alive, 6 months
93	Wu Tianhui (2002) (56)	25	F	L	20x20x18	>30	NO	RM	NET	Alive, 1 year
94	Chen Yaokun (2001) (57)	44	F	R	15x12x10	NA	NO	SM+RT	Local and systemic recurrence (lung)	Dead, 52 months later
95	Chen Yaokun (2001) (57)	34	F	R	12x10x10	NA	NO	RM	NET	Alive, 12 years
96	Zhang Renya 1994 (58)	45	F	L	2.5x2x1.5	>5	NO	SM	NET	Aline 6 months
97	Wu peijin 1995 (59)	26	F	L	8x6x5	NA	NO	SM+CT	Systemic recurrence	Dead, 48 months later
98	Wang Jin (2013) (60)	42	F	L	2x1.2x1	4	NO	WLE+CT	NET	Alive, 11 years
99	Wu Yongjun (1996) (61)	49	F	R	6x5.x3.5	>10	NO	SM	NET	NA
100	Gu Huaping (2005) (62)	54	F	R	7.5x1.2x6	>10	NO	SM	NET	Alive 10 months
101	Samenova (2023) (63)	45	F	R	10.5	NA	Axillary lymph nodes	Excision +CT	NET	Alive, 5 years
102	Sethi (2024) (64)	37	F	L	17x17x8	NA	NA	SM+CT	Local recurrence	Dead, 3 months later
103	Catarina Félix (2018) (65)	48	F	R	5.5x4.3.5	NA	NO	Palliative care	Local and systemic recurrence (liver, gallbladder, and pancreatic)	Alive, 6 years
104	Miyazaki C (2019) (66)	52	F	L	10	10	Several ipsilateral axillary lymph nodes	Neoadjuvant CT +SM	NET	Alive, 18 months
105	Galama (2021) (67)	87	F	L	8x4.4	NA	NO	SM+RT	NET	Alive, 20 months
106	Miroslav Lesar (2003) (68)	62	F	L	NA	NA	NA	RM+RT	NA	NA

cm, centimeter; CT, chemotherapy; F, female; HPF, high-power fields; L, left; M, male; MRM, modified radical mastectomy; NA, not available; NET: No evidence of tumor; R, right; RM, radical mastectomy; SM, simple mastectomy; RT, radiotherapy; WLE, wide local excision.

### Baseline characteristics of breast leiomyosarcoma patients

The clinical characteristics of 106 cases are listed in **Table 2**. The average age of the patients was 50.9 ± 14.4 years old, ranging from 18–87 years. There were 92 female cases, accounting for 86.8% of patients and the male cases accounted for 13.2%. 50.9% of patients had their primary tumor located on the left side. The average size of the primary tumor was 6.38 ± 4.98 cm. At the initial diagnosis, most patients (55.7%) have no lymph node or vascular invasion. Surgical treatment is the main treatment method for patients with primary fibrosarcoma of the breast, accounting for a high proportion 98.1%, including wide local excision (WLE), radical mastectomy (RM), simple mastectomy (SM) and modified radical mastectomy (MRM). The mean overall survival was about 34.53 months.

**Table 2:** The clinical characteristics of 106 cases.

Characteristics	n (%)
Age (mean ± SD, years)	50.9 ± 14.4
Gender	
Male	14(13.2%)
Female	92(86.8%)
Tumor location	
Left breast	54(50.9%)
Right breast	45(42.5%)
Left and right breast	1(0.9%)
Not mentioned	6(5.7%)
Tumor diameter (mean ± SD, cm)	6.38 ± 4.98
Lymph node/Vascular invasion	
Yes	5(4.7%)
No	59(55.7%)
Not mentioned	42(39.6%)
Treatment	
Surgery	89(84.0%)
No surgery	2(1.9%)
Surgery combined with radiotherapy and/or chemotherapy	15(14.1%)
Distant metastasis	
Yes	18(17.0%)
No	80(75.5%)
Not mentioned	8(7.5%)
Overall survival (mean ± SD, months)	34.53 ± 41.49

### Survival outcomes

A total of 98 patients were included in the OS analysis (**Figure 3**). The clinical characteristics of 98 cases are listed in **Table 3**. There were 84 female cases, accounting for 85.7% of patients and the male cases accounted for 14.3%. 51.0% of patients had their primary tumor located on the left side. Similarly, Surgical treatment is the main treatment method for primary breast fibrosarcoma patients.

**Table 3:** Clinical characteristics and cox multivariate regression analysis of 98 cases.

Characteristics	n (%)	HR (95%CI)	*P*
Tumor location		1.070 (0.425-2.693)	0.886
Right	39(39.8%)		
Left	50(51.0%)		
Right + Left	1(1.0%)		
NA	8(8.2%)		
Gender		1.286 (0.151-10.979)	0.818
Female	84(85.7%)		
Male	14(14.3%)		
Age (years)		0.301 (0.088-1.032)	0.056
≤37	15(15.3%)		
>37	83(84.7%)		
Tumor diameter		4.514 (1.146-17.784)	0.031
≤7cm	75(76.5%)		
>7cm	22(22.4%)		
NA	1(1.1%)		
Treatment		1.134 (0.869-1.479)	0.355
SM	28(28.6%)		
MRM	25(25.5%)		
Excision	13(13.3%)		
RM	7(7.1%)		
CT	1(1.0%)		
Surgery combined with RT and/or CT	14(14.3%)		
Simple tumor resection	4(4.1%)		
WLE	6(6.1%)		

CT, chemotherapy; NA, not available; RM, radical mastectomy; RT, radiotherapy; SM, simple mastectomy; WLE, extensive local excision.

**Figure 4:**
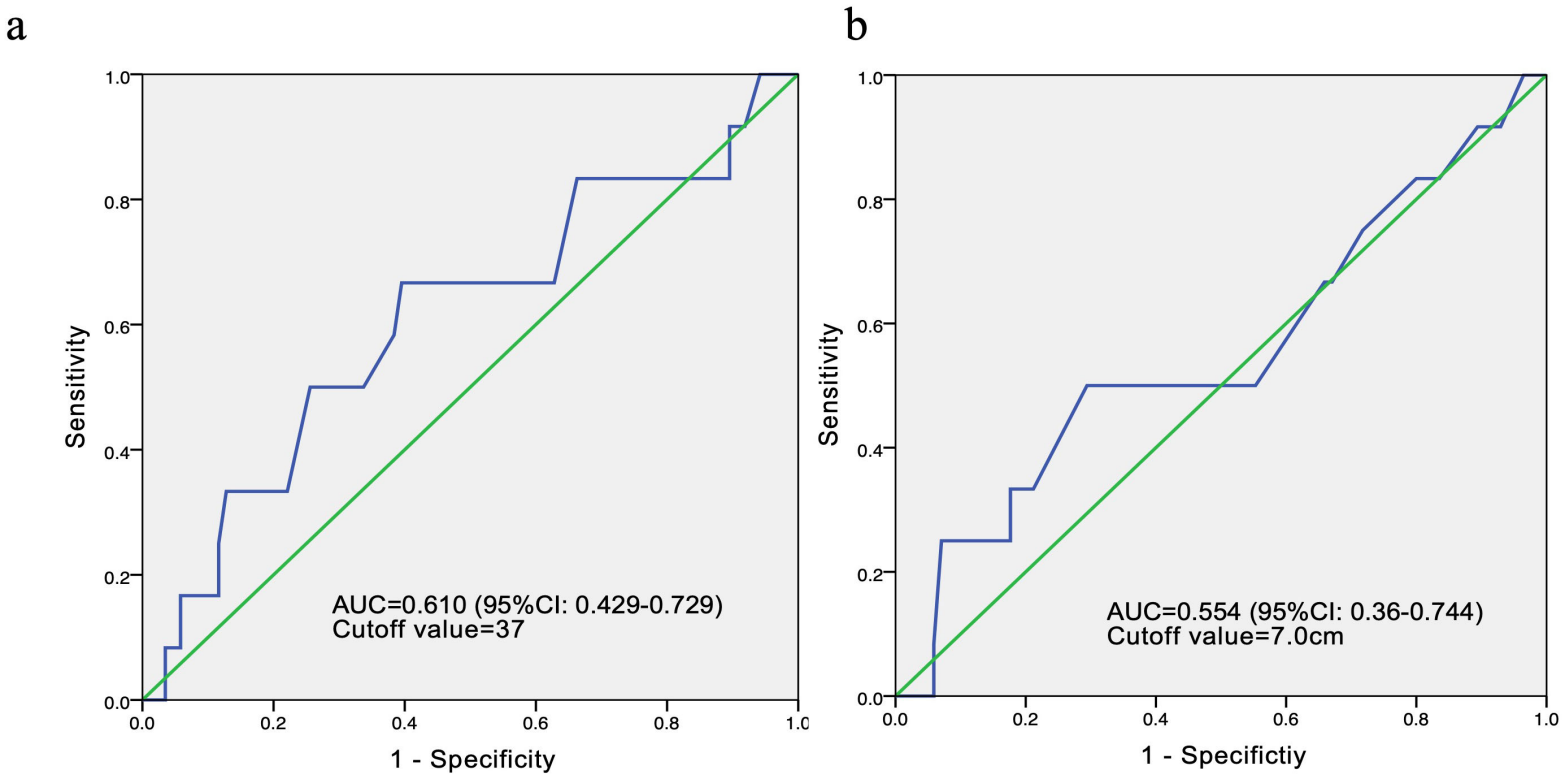
ROC curves for age **(a)** and tumor diameter **(b)** in 98 patients.

In order to conduct survival and Cox multivariate analysis, we use the ROC curve to calculate the cutoff values for age and the maximum diameter of the tumor. The results showed that the cutoff value for age was 37 years, with an area under the curve (AUC) of 0.610 and a 95% confidence interval of 0.429-0.729. The sensitivity and specificity are 0.667 and 0.86, respectively. The cutoff value for the maximum diameter of the tumor was 7cm, with an AUC of 0.554 and a 95% confidence interval of 0.360-0.744 (**Figure 4**). The sensitivity and specificity are 0.5 and 0.318, respectively.

**Figure 5:**
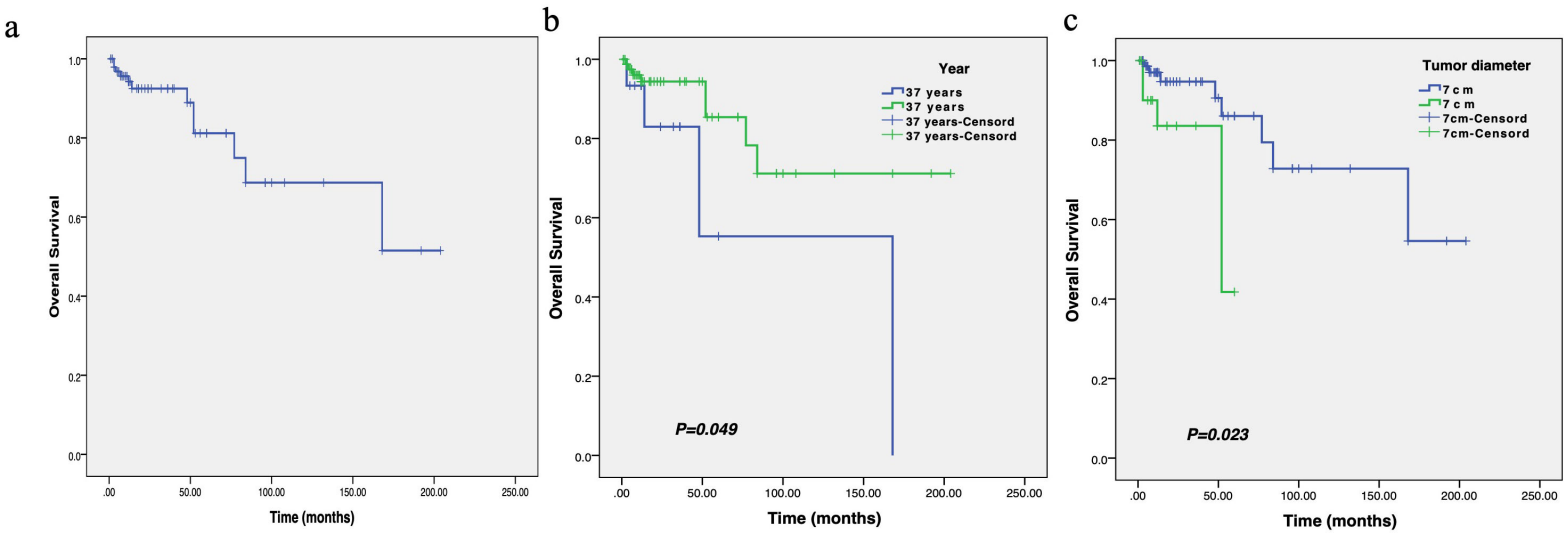
The survival curve of overall survival in patients with breast leiomyosarcoma **(a)**, as well as the comparison of overall survival between different ages **(b)** and tumor diameters **(c)**.

KM survival indicated that the median OS of these patients was 18.0 months (**Figure 5**). In subgroup analysis, patients with age ≤ 37 years at initial diagnosis or tumor diameter >7 cm before treatment had a shorter OS, and the differences were statistically significant.

### Prognostic factors from Cox regression

Cox proportional hazards univariate regression analysis consisted of data on tumor diameter, tumor location, patient age, gender and treatment method in 98 patients (**Table 3**). The results showed that the tumor diameter ≤ 7 cm could significantly improve the prognosis of patients with breast leiomyosarcoma (HR 4.514, 95% CI 1.146-17.784, *P* = 0.031).

Although there were differences in OS between subgroups aged over 37 and under 37 years, there was no statistically significant difference between age and OS in multivariate analysis (HR 0.301, 95% CI 0.088-1.032, *P* = 0.056). Whereas, the patient gender (HR 1.286, 95% CI 0.151-10.979, *P* = 0.818), tumor location (HR 1.070, 95% CI 0.425-2.693, *P* = 0.886) and treatment method (HR 1.134, 95% CI 0.869-1.479, *P* = 0.355) did not have a significant effect on the OS of breast leiomyosarcoma.

## Discussion

Breast sarcoma is a rare non-epithelial malignant tumor originating from the mesenchymal tissue of the breast, with approximately 4.6 new cases per million women per year, accounting for less than 1% of all breast malignancies (69, 70). Same with other soft tissue sarcomas, primary breast sarcomas are associated with genetic disorders such as familial adenomatous polyposis and neurofibromatosis type 1 (71). Risk factors include a history of radiotherapy, chronic lymphoedema, vinyl chloride exposure and Epstein-Barr virus (EBV) infection (72). Leiomyosarcomas are rare one of the subtypes to which it belongs, and its exact origin is unclear. It may develop from mesenchymal cells or smooth muscle cells within blood vessels and is most likely to occur in the vascular and muscular tissues of this anatomical region near the areola (1).

The clinical presentation of breast leiomyosarcoma is often a slow-growing large palpable mass, painless, firm, and lobulated, typically found in postmenopausal women (3). There is a tendency for skin and muscle invasion, but areola changes and nipple discharge are relatively rare (6). It is difficult to distinguish from other breast tumors in clinical practice because physical examination and imaging results are often similar to other malignant tumors (43), and are often mistaken for benign causes (lobular tumors and fibroadenomas) (8), and the diagnosis can only be finally confirmed through histological examination and immunohistochemical analysis after biopsy. Histopathology showed marked cellular heterogeneity, atypical mitoses, vascular invasion and necrosis (1). Immunohistochemistry demonstrated that leiomyosarcoma staining positive for desmin, smooth muscle actin and vimentin, whereas it was negative for epithelial markers, cytokeratin and S-100 (43, 73, 74).

Currently, there are insufficient guidelines for the treatment of breast leiomyosarcoma, probably due to the rarity of the disease in this location. As a result, the diagnostic and therapeutic approaches to this type of tumor are highly heterogeneous and require more specific treatment strategies and guidelines (43, 75). Because of the high rate of local recurrence, surgery with adequate resection margins is the only potential treatment for patients with sarcomas. A previous study showed that for optimal efficacy, a minimum negative margin of 3 cm should be achieved; however, a 2 cm margin can be used for breast protection (76). Several studies have reported metastatic spread to lungs, liver and bone, with lymph node involvement being extremely rare (5, 43, 66).

Our study also confirms that there are very few patients with primary breast leiomyosarcoma who are initially diagnosed with lymph node metastasis or vascular invasion. Routine lymph node dissection and sentinel lymph node biopsy is not recommended as it has no impact on patient survival (77). However, biopsy should be performed if lymph node metastasis is suspected on imaging. After surgical resection, radiotherapy is recommended for local control. Adjuvant radiotherapy after breast-conserving mastectomy has been shown to improve disease-free survival and local control of recurrence, especially if resection margins are inadequate (72). Chemotherapy may be indicated for tumors larger than 5 cm, high-grade tumors or advanced cancers (8).

Research suggests that while some patients with leiomyosarcoma (LMS) may benefit from immune checkpoint inhibitors (e.g., PD-1/PD-L1 inhibitors like nivolumab and pembrolizumab) (78), histological subtype analyses reveal that LMS has the lowest response rates compared to subtypes such as alveolar soft part sarcoma and undifferentiated pleomorphic sarcoma, which show the highest (78). Studies on combining these agents with chemotherapy (e.g., doxorubicin, dacarbazine) or radiotherapy in advanced LMS have demonstrated limited but promising clinical activity, with efficacy potentially dependent on the tumor’s immune microenvironment characteristics (79). It is hypothesized that by modulating this microenvironment, immunotherapy may convert immunologically “cold” tumors into “hot” ones, thereby promoting immune cell infiltration. Recent findings highlight that interactions between small venous smooth muscle cells and endothelial cells in breast tumors are critical for the infiltration of immune cells (e.g., T cells, B cells), suggesting a potential target for enhancing immunotherapy efficacy (80).

Current research directions primarily focus on combining immunotherapy with chemotherapy, targeted therapy, and radiotherapy. For instance, chemotherapy may create a favorable context for immunotherapy by inducing immunogenic cell death. Novel strategies, including those using genetically engineered tumor cells, are also under investigation. Adjunctive approaches such as certain biological therapies (e.g., interferon, interleukin-2) and adoptive cell therapies aim to modulate the body’s immune response against tumors. However, specific application data for these therapies in breast leiomyosarcoma remain limited.

However, our research found that patients with tumors larger than 7cm have a worse prognosis. Does this mean that patients with tumors larger than 7 cm may need to receive additional treatment besides surgery, in addition to other high-risk factors. Although, it is unclear whether treatment is beneficial or has any impact on morbidity and mortality. The combination of anthracyclines with the addition of ifosfamide has been described as first-line chemotherapy (43). There is also emerging evidence to support the use of neoadjuvant chemotherapy for the treatment of metastatic disease, but the results remain uncertain (66).

Hematogenous spread is the most common mode of metastasis in leiomyosarcoma (43). Distant hematogenous metastases to bone, liver, lungs, central nervous system and spine reported in about 25% of cases, and usually detected after a latent period of 15–20 years (5, 74). In patients with metastatic disease, palliative chemotherapy or palliative surgery may be offered to slow disease progression and control local complications (43, 81). Patients with this malignancy have a relatively poor prognosis and a high risk of recurrence compared to other types of breast cancer, with 5-year disease-free survival rates ranging from 33%-52% (82), making frequent follow-up and monitoring for post-excision recurrence necessary.

Our study provides the first systematic evidence that surgical approach, gender, age, and tumor location are not significantly associated with the prognosis of primary breast leiomyosarcoma. In contrast, tumor size was identified as an independent predictor of survival. Consequently, initial tumor size may be considered a key factor in guiding treatment decisions and identifying patients at high risk of recurrence.

But, there a key limitation of this review stems from the inherent methodological constraints of the available primary literature, which predominantly comprises single case reports and small, retrospective case series. Our systematic quality assessment using the JBI tools confirmed these limitations, highlighting frequent deficiencies in standardized follow-up and comprehensive outcome reporting. Consequently, the generalizability and robustness of our pooled findings should be interpreted with caution.

## Data Availability

The original contributions presented in the study are included in the article/**Supplementary Material**. Further inquiries can be directed to the corresponding authors.
