# Supplementary material for: Primary breast leiomyosarcoma: prognostic factors and treatment outcomes – a systematic review and case report (1969–2023)
Source: Front Oncol. 2025 Oct 30;15:1662132. doi: 10.3389/fonc.2025.1662132 (PMC12611670; doi:10.3389/fonc.2025.1662132)
Supplement: Supplementary file 1 [file DataSheet1.pdf]

Detailed Search Strategies

Search was conducted from inception until December 31, 2023.

1. PubMed

Search Date: [*December 31, 2023*]

Search Query:

("leiomyosarcoma"[MeSH Terms] OR "leiomyosarcoma"[Title/Abstract] OR "LMS"[Title/Abstract]) AND ("breast"[MeSH Terms] OR "breast"[Title/Abstract] OR "mammary"[Title/Abstract])

2. Web of Science

Search Date: [*December 31, 2023***]]**

Search Query:

TS=((leiomyosarcoma OR LMS) AND (breast OR mammary))

3. China National Knowledge Infrastructure (CNKI)

Search Date: [*2023 年 12 月 31 日*]

Search Query:

SU=('平滑肌肉瘤' + '乳腺' + '乳房') OR TI=('平滑肌肉瘤' + '乳腺' + '乳房') OR AB=('平滑肌肉瘤' + '乳腺' + '乳房')

4. Wanfang Database

Search Date: [*2023 年 12 月 31 日*]

Search Query:

(主题:("平滑肌肉瘤") AND 主题:(("乳腺") OR ("乳房"))) OR (题名:("平滑肌肉瘤") AND 题名:(("乳腺") OR ("乳房")))
